# Influence of Overnight Orthokeratology Lens Treatment Zone Decentration on Myopia Progression

**DOI:** 10.1155/2019/2596953

**Published:** 2019-11-15

**Authors:** Anken Wang, Chenhao Yang

**Affiliations:** Department of Ophthalmology, Children's Hospital of Fudan University, Shanghai 201102, China

## Abstract

**Purpose:**

To investigate the effect of OK lens treatment zone decentration on myopia control.

**Methods:**

We retrospectively selected 30 OK lens wearers who met the following conditions in our hospital from more than 1300 cases: wearing lens in both eyes and only one eye was off-center while the other one was centric for more than 12 months. During the period of follow-up, the UCVA of each eye was better than 0.1 of logMAR and there were no obvious tropia, Kappa angle, and complications such as glare and diplopia.

**Result:**

Among 30 cases, 15 are males and 15 are females, with an average age of 9.3 ± 1.51*Y*. There were no significant differences in equivalent spherical lens, astigmatism, *e* value, flat *K*, steep *K*, astigmatism, lens diameter, and toric between the two groups (*p* > 0.05). The average distance of decentration was 0.73 ± 0.25 mm. Axis growth per year in was 0.20 ± 0.24 mm the OK-lens-decentered group and 0.29 ± 0.20 mm in the OK-lens-centric group, which shows significant difference between them (*p* < 0.05). According to the direction of decentration, 30 decentered eyes were divided into temporal group (20 eyes) and other direction group (10 eyes). The efficiency of myopia control (the growth of AL per year in OK-lens-decentered eye/the growth of AL per year in the contralateral OK-lens-centric eye) was 0.69 ± 0.50 in the temporal decentration group and 0.75 ± 0.52 in the other direction group, showing no significant difference between them (*p* > 0.05). There was no significant correlation between the efficiency of myopia control and the degree of decentration among temporal decentration group (*p* > 0.05).

**Conclusion:**

This self-control study without much interference factors shows that the decentration of OK lens can delay the development of myopia more effectively than being centric when uncorrected visual acuity was acceptable without obvious corneal complications, glare, or ghosting.

## 1. Background

Globally, uncorrected refractive errors constitute the second major cause of vision loss of which myopia is the most common and well known [1, 2]. To date, it has been estimated that myopia currently affects approximately 30% of the world's population, and it has been forecasted that the number will rise to 50% by 2050 [3]. The prevalence of myopia in young adolescents is also increasing; for instance, there are about 70% of senior high school students in China who have been diagnosed as myopia nowadays [4]. Therefore, finding effective therapies to slow the progression of myopia could potentially benefit millions of individuals.

Modern orthokeratology (OK) is a clinical nonsurgical method for temporary myopia correction and even controlling myopic progress in adolescents [5–9]. With professional inspection, clinical monitoring, and careful personal hygiene management, the safety of overnight OK treatment has repeatedly been confirmed [10, 11]. Nowadays, orthokeratology is considered to be one of the most promising means of controlling the progress of myopia in children [12, 13]. The mechanism of myopia control is not completely clear, but it is generally believed that wearing OK lenses can reshape the anterior corneal surface, flatten the central cornea, steepen the paracentral cornea, change the image quality of the central and peripheral retina, and finally form the peripheral defocus [14, 15]. However, after orthokeratology, the center of the corneal optical area cannot be consistent with the pupil center in some patients. The decentration of the corneal plastic area may result in the increase of corneal irregular astigmatism and higher order aberration, which results in glare and other symptoms [16]. Previous reports mostly focused on its prevention and influence; whether the decentration can influence the myopia progression is rarely reported. The purpose of this study was to observe the decentration of optical zone after orthokeratology and its effect on controlling the myopia, so as to provide evidence for the mechanism of controlling myopia after orthokeratology.

## 2. Methods

### 2.1. Subjects

In this retrospective study, we reviewed all the patients who started orthokeratology between January 2015 and December 2017 treated at the Children's Hospital of Fudan University.

#### 2.1.1. Clinical Pathway

As the first visit, all the patients underwent comprehensive examination including cycloplegic refraction, uncorrected visual acuity (UCVA), best-corrected visual acuity (BCVA), extraocular movements, corneal light reflection test, intraocular pressure, slit-lamp examination, fluorescein staining, corneal endothelial cell density, axial length, dilated fundoscopy, and corneal topography. Appropriate prescriptions for OK lens were given to them by different experienced doctors, and the patients were asked to wear OK lenses no fewer than 8 h per night and visit subsequently every 3 months. At every follow-up afterwards, they took a detailed list of ocular examinations including corneal light reflection test, slit-lamp evaluation, fluorescein staining, axial length, UCVA, and corneal topography. All subjects were treated according to the tenets of the Declaration of Helsinki.

The inclusion criteria include the following: (1) The spherical refractive error must be less than −5.00 DS with a refractive astigmatism of −1.50 DC or less and BCVA of logMAR (logarithm of the minimum angle of resolution) must be 0.0 or better before treatment. (2) OK lenses must be worn in both eyes, but only one eye should be direction-sustained decentered more than 0.5 mm and less than 1.5 mm only vertically or horizontally while the contralateral eye maintained central location. Both the decentration and central location must maintain for more than 12 months and the amount of decentration must vary within 0.5 mm (determined by corneal topography in four continued visit). (3) The UCVA of each eye must be better than 0.1 (logMar) after removal of lens at each follow-up.

**Figure 1:**
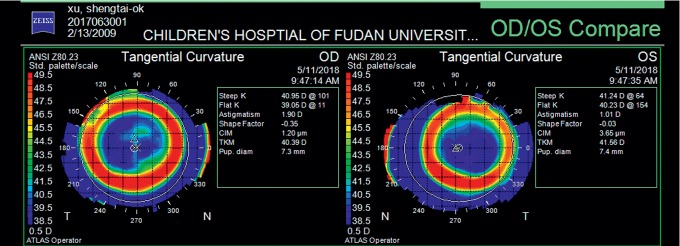
Corneal topography (tangent diagram) of an included subject as an example. *x* represents the center of corneal topography. Δ represents the corneal apex. ○ represents the pupil center.

The exclusion criteria include the following: (1) The subjects included should not have obvious corneal complications, glare, duplication, or any other symptoms (during each follow-up, we routinely asked children if they had glare, diplopia, or other symptoms and checked both the condition of corneal and lens care). (2) Subjects with underlying ocular disease such as retinopathy, prematurity, neonatal problems, history of genetic disease, neurodevelopment condition that might affect refractive development, or other system disorders associated myopia were excluded from this study. (3) Enrolled subjects could not have any amount of tropia by cover-uncover test at far (4.0 m) and near (0.33 m) or obvious Kappa angle.  Figure 1 is the corneal topography of an included subject as an example.

### 2.2. Measurements of Optical Parameter

Cycloplegic refraction was measured two times by specialized technicians to make sure of the exactness.

#### 2.2.1. Auto Refract Keratometer

*K* value was measured three times routinely with auto refract keratometer (NIDEK, Co; LTD, Japan. Model: ARK-1) by the same specialized technician, and then, the average value was recorded.

#### 2.2.2. IOL-Master

Axial length was measured three times routinely with IOL-Master 500 (Carl Zeiss Meditec, Ag. jena, Germany) by the same specialized technician, and then, the average value was recorded.

#### 2.2.3. Corneal Topography

Corneal profiles were measured with Carl Zeiss Atlas Corneal Topography System-9000 (Carl Zeiss Meditec, Inc. California, United States of America, Model 9000) by the same specialized technician. And each of the profiles was the best-focus image (the accuracy greater than 95%) from the four frames which were captured automatically. Either the equivalent *e* value, steep *K*, flat *K*, and corneal toricity at baseline or the corneal topography after orthokeratology can be measured precisely by using Placido rings to map the corneal surface and provide topography data.

### 2.3. Measurement of Treatment Zone Decentration

The amount of decentration of OK lens was measured by finding the distance between the center of the treatment zone and the pupillary center [17, 18].

**Figure 2:**
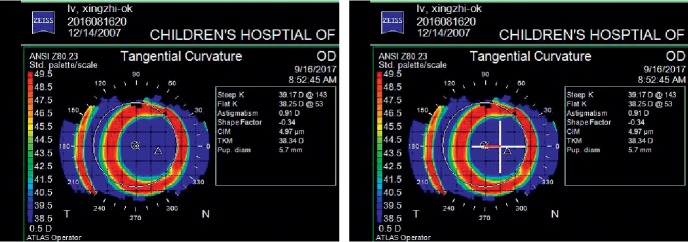
The measurement of treatment zone decentration. The length of the red line in the right part is the amount of decentration.

According to the corneal topography, treatment zones ranged from the corneal apex to where the keratometry values changed within 1 D and less than 2 types of colors in the palette scale. The center of the treatment zone after orthokeratology was determined by marking the farthest 4 edges of the optical zone of the corneal topography map in the vertical and horizontal direction by software (Photoshop 6.0) and then the intersecting point of these 4 points can be the center. The pupillary center was determined by corneal topography with pupil-finding software. The distance between the center of the treatment zone and the pupillary center was measured precisely by the ruler of the software compared with the grid (one grid stands for 1 mm). The way of measurement can be seen in Figure 2.

After the distance between the pupillary center and the center of the treatment zone was measured, the subjects whose decentration is upon 0.5 mm both vertically and horizontally were excluded. Only patients with a decentration larger than 0.5 mm for 12 months were included in the lens-decentration group; in addition, the decentration should be less than 1.5 mm, so that sclera would not interact with the lens.

### 2.4. Lenses

All OK patients were fitted with five‐zone reverse geometry OK lenses (*α* ORTHO‐K®; ALPHA Corp, Nagoya, Japan), with a nominal Dk of 104 × 10^−11^ (cm^2^/s) (mL O_2_/mL·mmHg) or (LUCID ORTHO‐K®, LUCID Corp, Fenghua County, Korea) with a nominal Dk of 100 × 10^−11^ (cm^2^/s) (mL O_2_/mL·mmHg) in accordance with the manufacturer's fitting instructions. After lens dispensing, patients were recommended to wear their OK lenses for at least eight consecutive hours every night. Upon stabilization of refractive error correction, they were instructed to wear their lenses for at least five nights per week. Refraction, visual acuity, corneal topography, and lens fitting were evaluated at every visit. The procedures for fitting, prescription, and replacement of OK lenses were all performed by experienced specialists.

### 2.5. Statistical Analysis

SPSS Statistics 24.0 (IBM Statistics, Armonk, NY) was used for statistical analysis of the lens fitting decentration and ocular biometric parameters. The Shapiro–Wilk test was used to check the normality. The difference of parameters and axial length growth between every pair of OK-lens-decentered (OLD) eye and OK-lens-centric (OLC) eye was compared using the paired *t*-test. The rate of axial growth was analyzed concerning lens fitting decentration by the Pearson correlation (*r*) test. *p* value less than 0.05 was considered statistically significant.

## 3. Results

Thirty subjects (15 males and 15 females) were evaluated with their mean age of 9.3 ± 1.51 (mean ± standard deviation, range 8 to 13 years). Before orthokeratology, the spherical equivalent refractive error, spherical refractive error, and regular astigmatism of the OLD eyes were −2.58 ± 1.15 D (range −1.00 to −4.75 D), −2.39 ± 1.05 DS (range −0.75 to −4.75 DS), and −0.44 ± 0.52 DC (range 0.00 to −1.50 DC), respectively, while those in the OLC eyes were −2.53 ± 1.16 D (range −1.00 to −5.00 D), −2.32 ± 1.06 DS (range −0.75 to −4.75 DS), and −0.50 ± 0.50 DC (range 0.00 to −1.50 DC), respectively. The difference between two groups was not statistically significant by paired *t* test in spherical equivalent refractive error (*p*=0.60), spherical refractive error (*p*=0.43), and regular astigmatism (*p*=0.35). Before orthokeratology, the steep *K*, flat *K*, corneal toricity, and equivalent *e* value of the OLD eye were 43.60 ± 1.42 D (range 39.75 to 46.00 D), 42.78 ± 1.33 D (range 40.00 to 45.00 D), 0.86 ± 0.53 D (range 0.25 to 2.25 D), and 0.61 ± 0.08 (range 0.42 to 0.77), respectively, while those in the OLC eye were 43.61 ± 1.65 D (range 39.25 to 46.25 D), 42.86 ± 1.56 D (range 39.50 to 46.00 D), 0.84 ± 0.54 D (range 0.00 to 2.25 D), and 0.63 ± 0.10 (range 0.42 to 0.85), respectively. The difference between two groups was not statistically different by paired *t* test in steep *K* (*p*=0.96), flat *K* (*p*=0.40), corneal toricity (*p*=0.80), and equivalent *e* value (*p*=0.15). The biological parameters of both eyes and data of their lenses can be seen in Table 1.

Among thirty pairs of eyes, there were three subjects who wore different grands OK lens in each eye, but the Wilcoxon signed rank test shows no statistical difference between two groups (*p*=0.102). The lens diameter and lens toricity were 10.59 ± 0.19 mm (range 10.00 to 11.00 mm) and −0.17 ± 0.38 D (range −1.00 to 0.00 D) in the OLD group, while 10.61 ± 0.17 mm (range 10.20 to 11.00 mm) and −0.12 ± 0.36 D (range −1.50 to 0.00 D) in the OLC group, showing no statistical difference by the paired *t* test (*p*=0.184 and *p*=0.326).

**Figure 3:**
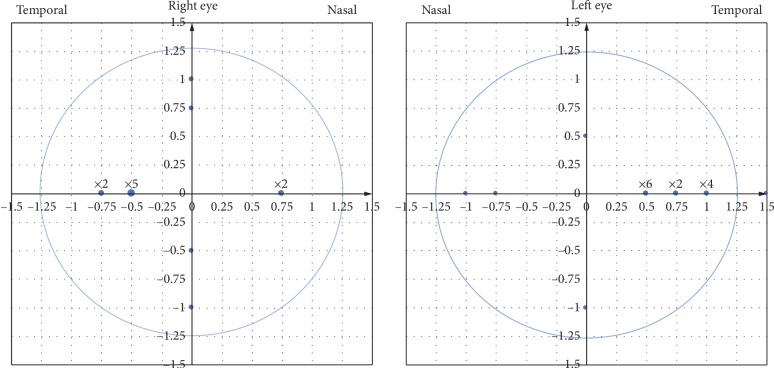
The position of lens fitting among 30 OLD eyes. Each lattice represents 0.25 mm on corneal and the symbol of ×2 means there are two cases of this position, and so on.

Distance of lens fitting decentration in the OLD group was 0.73 ± 0.25 mm (range 0.50 to 1.50 mm), including 20 (66.67%) temporal, 4 (13.33%) nasal, 3 (10%) superior, and 3 (10%) inferior (Figure 3). The distance of decentration shows no statistical correlation with its direction by the Spearman rank correlation coefficient test (*p*=0.165) and no statistical correlation with the spherical equivalent refractive error (*p*=0.65), corneal toricity (*p*=0.40), equivalent *e* value (*p*=0.96), lens toricity (*p*=0.27), and lens diameter (*p*=0.99) by Pearson correlation coefficient test.

**Figure 4:**
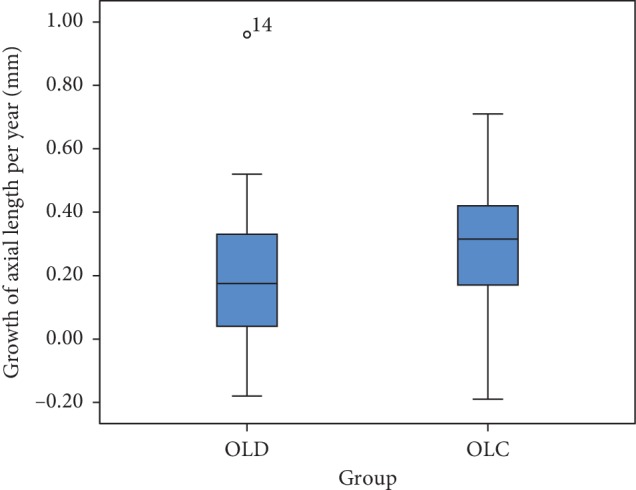
Box plot for comparison about mean axial length growth per year between OLD group and OLC group.

Mean axial length growth per year in OLD group is 0.20 ± 0.24 mm and 0.29 ± 0.20 mm in the OLC group. Statistically significant difference was found in mean axial length growth per year between OLD and OLC group by the paired *t* test (*p*=0.003) (Figure 4).

Because the major direction is temporal, while the other three directions are too few for statistical analysis, we divided all OLD eyes into temporal group and other direction group. To avoid bring individual differences, it is inappropriate to compare the amount of axial growth directly among different children. Thus, we use the ratio of axial growth rate between paired OLD eye and OLC eye to express the efficiency of myopia control (EMC: growth of AL per year in OLD eye/growth of AL per year in the contralateral OLC eye) instead of value of axial growth. The average EMC is 0.69 ± 0.50 in the temporal decentration group and 0.75 ± 0.52 in the other direction group, which shows no statistical difference by two independent samples *t*-test (*p*=0.75). The mean magnitude of decentration is 0.70 ± 0.28 mm in the temporal group and 0.80 ± 0.20 mm in the other direction group, showing no statistical difference by two independent samples *t*-test (*p*=0.27), which eliminates the interference caused by the different degrees of decentration between two groups.

**Figure 5:**
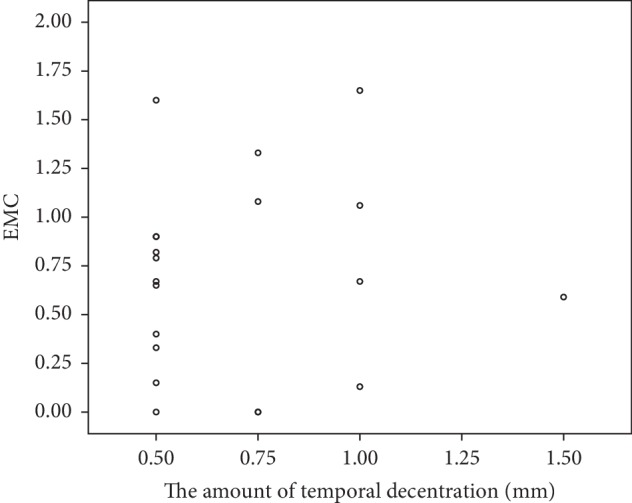
Scatter plot for relationship between EMC and the amount of decentration in 20 eyes with temporal decentration.

20 eyes with temporal decentration were selected for analyzing the relationship between the EMC and the amount of decentration. The average EMC is 0.69 ± 0.50 and the average decentration is 0.70 ± 0.28, and there is no statistical relationship between them by the Pearson correlation analysis (*p*=0.75) (Figure 5).

## 4. Discussion

Owing to the effectiveness of controlling myopia in adolescents, there is a gradual increase in application of OK treatment that has been chosen by more than 1.5 million adolescents in China [19]. However, fitting decentration cannot be completely avoided during the whole procedure. In the clinical application of orthokeratology, lens decentration has become a common problem for both physicians and patients, probably nearly to half percent according to some study [16, 20, 21]. Since the off-center of OK lens is so common, whether it can affect the progression of myopia or not aroused the authors' interest and attention.

Whether the decentration of OK lens makes difference on myopia controlling is a fresh and difficult question to solve because it is hard to measure the decentration and to exclude the confounding factors especially individual differences like age, ocular biometric parameters, eye care habits, daily exposure of sunshine outdoor, genetic characteristics, and the frequency of wearing spectacles or lens.

About the measurement of decentration, as far as we are concerned, it can be the distance between the center of the treatment zone and the initial corneal apex, but it will take a lot of efforts to make the difference maps on corneal topography between the new one and initial one for all the patients. It can also be the distance between the center of the treatment zone and the pupillary center which is used by many other reports [17, 18, 22]. In the latter one, just a tangential map at that time is needed and the influence of Kappa angle can be reduced, so we pick the latter one.

About the results, Wu et al. recently found that the decentration of OK lens will slow down the growth of myopia by retrospectively analyzing right eyes of 134 children wearing OK lens decentered in varying degrees [22]. It is an elaborate research but still cannot avoid the individual differences. In our study, we investigated the sole influence of overnight OK lens fitting decentration on myopia progression by self-control study, that is to say, found the subjects who wore OK lens in both eyes while single OK-lens was decentered, excluding most of the individual differences, differing from the previous study. In this research, we found that for the same child, the axial length of the OLD eye grew slower than that in the OLC eye according to the usual definition of decentration [17, 18], which is similar to Wu's result. Of course, this is on the premise that the decentration is less than 1.5 mm and the glare, ghosting, or corneal epithelial staining is not obvious. Therefore, according to the result, since the decentration of OK lens is hard to avoid completely in our clinical practice, we need not worry about the adverse effect of off-center on myopia control too much if the vision of children is acceptable; the amount of decentration is not very serious and there is no obvious complications with the child.

The reason of decentration has always been a hot topic. Chen found that the magnitude of corneal asymmetry vector significantly contributed to the amount of lens decentration, whereas the baseline spherical or astigmatic refractive error, corneal eccentricity, flat *K*, horizontal visible iris diameter, or lens diameter did not affect orthokeratology lens decentration [18]. In our research, there is no significant difference in corneal asymmetry or other ocular parameters between two eyes, so why did the continued condition occur in which one eye is in right position while another is decentered ? According to our conjecture, it may attribute to the different strength of eyelid between two eyes and the doctor's prescription for lens. The strength of eyelid is hard to measure or control, and for that reason, it is rarely reported. The reason why the decentration is monocular is still unknown, promoting us to continue further study.

It is confirmed that temporal is the most frequent direction of decentration (horizontal and vertical); for example, Chen found 84.9% temporal in 106 OK-lens-decentered eye and Yang found 48.5% temporal in 270 OK-lens-decentered eye which is the most common direction [20, 21], and our results are in relatively good agreement with those of previous reports.

It will lead to errors caused by large individual differences if we use the axial length values directly when exploring the relationship between AL growth and direction or degree of decentration among different children, so we introduced the new variable named EMC, which shows how strong the effect of decentration on myopia control is in the same child. In previous comparative analysis of OLD eyes and OLC eyes, we used paired *t* test because they are from the same child and had the almost same parameters, which can reflect the difference caused by different positions more accurately, but when analyzing EMC, two independent sample *t* tests were used because the subjects in OLD group were from different children. According to the analysis, whether the decentration is temporal or not does not affect the EMC on the premise that the interference from its different degrees has been eliminated. Unfortunately, it is not appropriate to discuss all directions in detail, because the cases with not-temporal-directions decentration are too few for statistical analysis. At the same time, from the analysis of 20 eyes with temporal decentration, we found there was no statistical correlation between EMC and the degree of decentration when the distance is over 0.5 mm, while Wu's study showed the opposite result [22]. Because of the design of this retrospective experiment, the sample size for this problem is relatively small, so we need many large sample studies to confirm this conclusion.

Although orthokeratology has been proved to be effective in controlling myopia, it is mechanism is still not totally clear. The most possible one is considered the peripheral defocus forced by reshaping the anterior corneal surface and then changing the image quality of the central and paracentral retina affecting the axis growth by pathway of choroid, sclera, etc [23]. One possibility is that a dose-response relationship exists where greater amounts of peripheral myopic defocus result in greater reductions in myopia progression. Another possibility is that there is a range-response relationship and that any amount of myopic peripheral defocus above some threshold acts as a “stop” signal to slow myopia progression. If range-response or dose-response relationship exists, the greater reductions in myopia progression in OLD eyes are possible as more peripheral locations may experience the myopic defocus or more total peripheral myopic defocus may be formed when the pupil is closer to the edge of treatment zone [24]. Up to now, there are rare research studies mentioning the measurement of the peripheral defocus accurately when the lens is off-center. In addition, testing the peripheral defocus was limited by the lack of instruments, so it is hard to estimate the difference of the range and degree of peripheral defocus between OLD eyes and OLC eyes. It is worthwhile for us to do further research about peripheral defocus.

In this study, 6 eyes were found to have different degrees of axial shortening. On the one hand, it may be caused by measurement error; on the other hand, the most possible reason is the increase of choroidal thickness after orthokeratology and other scholars have also found some similar cases of shortened axial length [25, 26]. As it is known to all, IOL-Master measures the length between the corneal vertex and retinal pigment epithelium along the visual axis using a red fixation beam, which is the most common way to measure the axial length, so the thicker choroid will lift the retinal pigment epithelium and decrease the axial length measured according to the theory [25, 26]. However, the relationship between formation of thicker choroid and peripheral myopic defocus remains to be studied, so as the question about whether the increase of blood flow in thicker choroid affects the progression of myopia.

One of the limitation of this study is that the sample size is small especially the case with not-temporal-direction decentration. But to be honest, it is very difficult to find 30 cases that meet the inclusion and exclusion criteria from more than 1300 cases. Another limitation is we did not measure the peripheral defocus precisely, which may prompt us to move on to a more complete prospective study.

## 5. Conclusion

To summarize, lens decentration is a common phenomenon in orthokeratology. By excluding most of the interference factors, we found that OK lens being decentered less than 1.5 mm can delay the development of myopia more effectively than being centric when UCVA was acceptable without obvious corneal complications, glare, or ghosting. And there was no significant correlation between the effect and the degree or direction of decentration. But we still do not recommend the intention to make the lens decentered by modifying original prescription, because the complications still can be intractable problems.

## Figures and Tables

**Table 1:** Difference of biological parameters and lens between OLD eyes and OLC eyes.

Parameter (mean ± SD)	OLD eye (*n* = 30)	OLC eye (*n* = 30)	*p* value
Spherical equivalent refractive error (D)	−2.58 ± 1.15	−2.53 ± 1.16	0.60
Spherical refractive error (DS)	−2.39 ± 1.05	−2.32 ± 1.06	0.43
Regular astigmatism (DC)	−0.44 ± 0.52	−0.50 ± 0.50	0.35
Equivalent *e* value	0.61 ± 0.08	0.63 ± 0.10	0.15
Steep *K* (D)	43.60 ± 1.42	43.61 ± 1.65	0.96
Flat *K* (D)	42.78 ± 1.33	42.86 ± 1.56	0.39
Corneal toricity (D)	0.86 ± 0.53	0.84 ± 0.54	0.80
Lens toricity (D)	−0.17 ± 0.38	−0.12 ± 0.36	0.37
Lens diameter (mm)	10.59 ± 0.19	10.61 ± 0.17	0.18

## Data Availability

The raw data used to support the findings of this study are included within the supplementary materials.

## Supplementary Materials

Supplementary Materials The excel sheet can be described as “ocular biometric parameters of 30 children included in this study before orthokeratology and the growth of AL one year after.
